# Progress in Medical Education

**Published:** 1893-10

**Authors:** 


### Progress in Medical Education.

President Eliot, of Harvard University, in his speech to the Alumni on Commencement Day, announced that the Law School had raised its standard by requiring, after the year 1896, the degree of A.B., S.B., or Ph. B., as a prerequisite for admission to its courses. There is already such a requirement in the Divinity School, and President Eliot expressed the hope that hereafter it would be adopted for the Medical School.

